# Apical periodontitis microbiome association with salivary and serum inflammatory burden

**DOI:** 10.1111/iej.14184

**Published:** 2025-01-04

**Authors:** Abdulaziz Bakhsh, Susan Joseph, Francesco Mannocci, Gordon Proctor, David Moyes, Sadia Ambreen Niazi

**Affiliations:** ^1^ Department of Restorative Dentistry, Division of Endodontics, Faculty of Dental Medicine Umm Al‐Qura University Makkah Kingdom of Saudi Arabia; ^2^ Centre for Host‐Microbiome Interactions, Faculty of Dentistry, Oral & Craniofacial Sciences Guy's Dental Hospital, King's College London London UK; ^3^ Department of Endodontics, Centre of Oral Clinical and Translational Sciences, Faculty of Dentistry, Oral and Craniofacial Sciences Guy's Dental Hospital, King's College London London UK

**Keywords:** apical periodontitis, inflammatory markers, microbiome, targeted 16S rRNA sequencing

## Abstract

**Aims:**

Apical Periodontitis (AP) involves complex interactions between the root canal microbiome and the host immune response, with potential risk of local and systemic inflammatory burden, however there is no evidence available regarding correlation between microbiome and inflammatory marker levels. This study aims to identify the microbiome of saliva, intracanal and blood samples in AP subjects and investigate the correlation between intracanal and blood microbiomes with serum inflammatory biomarker levels, and salivary microbiomes with salivary inflammatory biomarker levels.

**Methodology:**

Saliva, Intracanal and blood samples were collected from AP patients undergoing root canal retreatment. Following DNA extraction, 16SrRNA gene‐sequence analysis (V1–V2) was performed using Illumina MiSeq 300 platform. Serum and salivary inflammatory marker levels were measured using the magnetic multiplex‐microbead assay. The alpha and beta diversities were tested using the phyloseq package in R (version 4.1). The abundance of the identified phyla and genera were analysed using non‐parametric tests. Spearman´s correlation coefficient was used for correlation between microbial abundance and biomarker levels.

**Results:**

Streptococcus and Prevotella were prevalent in saliva; Enterococcus, Streptococcus and Bacteroidaceae_(G‐1) in intracanal; and Cutibacterium and Staphylococcus in blood samples. Streptococcus, Prevotella, Actinomyces and Rothia were the most abundant common genera among all three sample sources. In saliva, Haemophilus, Gemella, Prevotella, and Alloprevotella were positively correlated with salivary levels of IL‐8, MMP‐2, TNF‐α, and IL‐6, respectively. Intracanal genera, Enterobacter, and Parvinomonas, were positively correlated serum FGF‐23. Finally, the abundance of Novosphingobium, Streptococcus, Bosea, and Corynebacterium genus in blood were positively correlated with FGF‐23, MMP‐9, CRP, IL‐8, and ICAM‐1.

**Conclusion:**

Microbiome in saliva, blood and intracanal samples were correlated with some of the inflammatory biomarker levels of saliva and serum, suggesting that the effect of AP goes beyond a periapical infection and may pose a potential systemic inflammatory burden risk if left untreated.

## INTRODUCTION

Apical periodontitis (AP) results from microbial invasion of the root canal system causing periapical tissue destruction, necessitating root canal treatment (RCT). Persistent root canal infections can arise from inadequate disinfection during RCT, often requiring retreatment or surgery (Aw, 2016). Studies using culture or molecular‐based approaches have revealed that the microbiome of these infections is diverse (Niazi et al., 2010, 2016; Siqueira Jr. & Rôças, 2022; Sun et al., 2022). Recent molecular techniques showed that Gram‐negative microbes like those found in saliva are also present in this diverse root canal microbiome, suggesting a potential link between salivary and root canal bacteria (Zandi et al., 2018).

AP involves complex interactions between the root canal microbiota, virulence of bacteria, and the host immune response, resulting in increased local and systemic inflammatory markers. Elevated systemic inflammatory biomarkers are associated with cardiovascular diseases (CVDs) risk factors and may contribute to endothelial dysfunction leading to atherosclerosis (Niazi & Bakhsh, 2022). Our previous research indicated that AP is associated with elevated systemic inflammatory markers levels, including Interleukin (IL)‐1β, high‐sensitive C‐reactive protein (hs‐CRP), Fibroblast growth factor (FGF)‐23 and asymmetric dimethylarginine (ADMA), which decreased after successful endodontic treatment (Al‐Abdulla et al., 2023; Bakhsh et al., 2022).

This study aimed to characterize the microbiomes of saliva, intracanal and blood samples from AP subjects and investigate potential microbiome similarities amongst these sources. It also explored differences in the intracanal microbiome between symptomatic and asymptomatic AP cases. Additionally, the study examined associations between intracanal and blood microbiomes with serum inflammatory biomarker levels, as well as salivary microbiomes with salivary inflammatory biomarker levels, as previously investigated (Bakhsh et al., 2022, 2023).

## MATERIALS AND METHODS

### Patient recruitment and sample collection

The protocol was approved by the London‐Hampstead Research Ethics Committee (IRAS project ID 207795). Written consent was obtained in accordance with the Declaration of Helsinki. Subjects with AP (*n* = 65) requiring endodontic treatment in the post‐graduate endodontic consultation clinic at Guy's Hospital were recruited. Detailed medical/dental history and clinical/radiographic examinations were recorded and suitable subjects were selected based on the inclusion criteria as outlined in our previous study (Bakhsh et al., 2022). Sample collection procedures are explained in the Data S1.

### Inflammatory biomarker analysis

Serum and salivary inflammatory biomarkers were analysed in our previous studies (Bakhsh et al., 2022, 2023). Serum and Saliva samples were collected from recruited subjects. Levels of inflammatory markers Interleukin (IL)‐1β, IL‐6, IL‐8, tumour necrosis factor (TNF)‐α, pentraxin‐3, intercellular adhesion molecule (ICAM)‐1, vascular cell adhesion molecule (VCAM)‐1, high‐sensitive C‐reactive protein (hs‐CRP), fibroblast growth factor (FGF)‐23, E‐Selectin, matrix metalloproteinase (MMP)‐2, MMP‐8, MMP‐9 were then measured using the magnetic multiplex microbead assay according to the manufacturer's instructions of each analyte. Complement 3 (C3) was measured using the enzyme linked immunosorbent assay according to manufacturers' instructions.

### Bacterial DNA extraction and quantification

The bacterial DNA from saliva and intracanal samples was extracted using an optimized two‐day extraction protocol (Dore et al., 2015), whilst from blood samples, the QIAmp® blood mini kit (Qiagen, Venlo, Netherlands) was used according to manufacturer's instructions. Extracted DNA was quantified using the Qubit® 3 Fluorometer (ThermoFisher Scientific) according to the manufacturer's instructions.

### Targeted 16S rRNA gene sequencing

#### Preparation of 16S rRNA gene amplicon libraries for sequencing on the Illumina MiSeq platform

**FIGURE 1:**
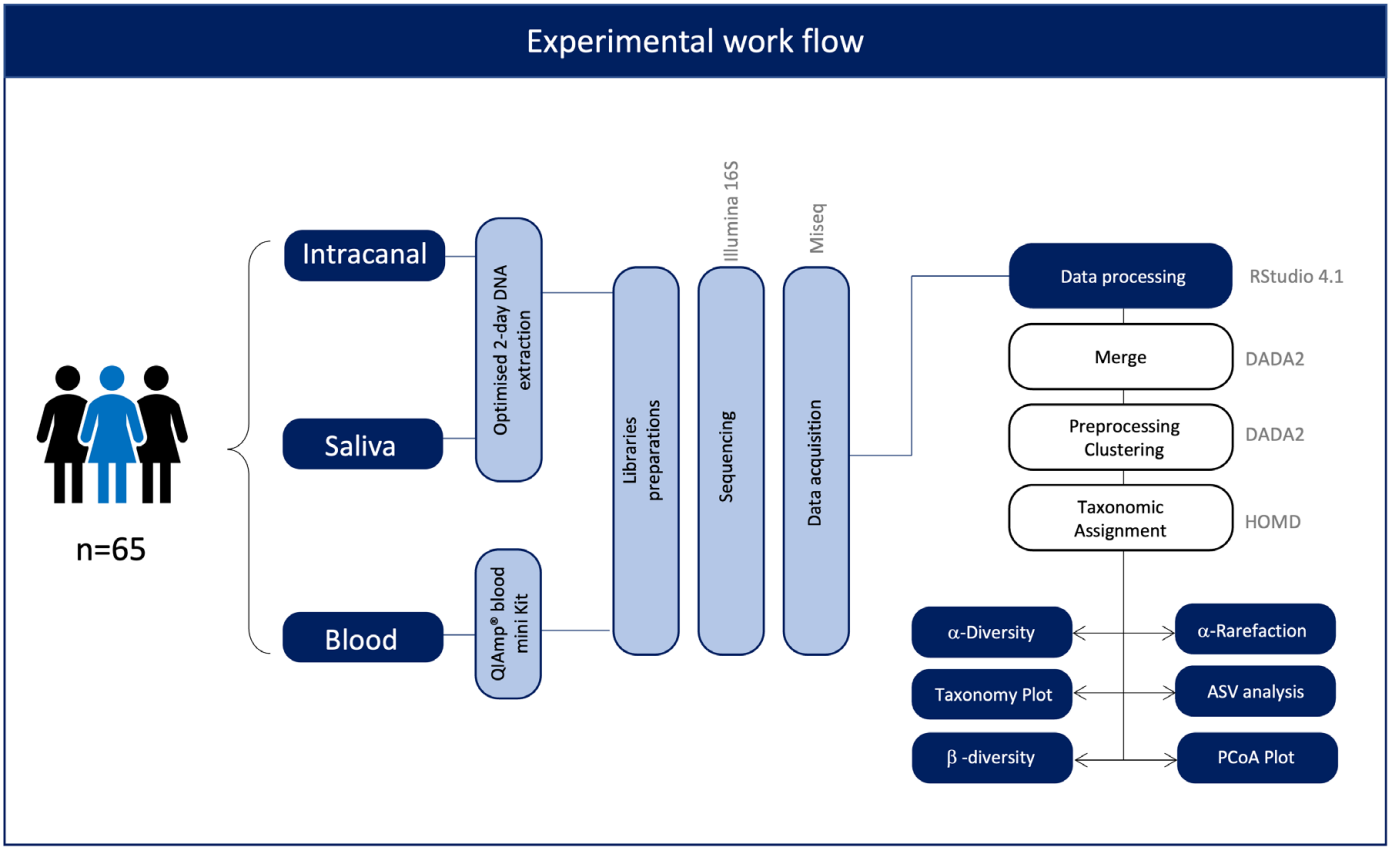
Experimental workflow of collected samples. HOMD: Human oral microbiome database; ASV, Amplicon sequence variant; PCoA, Principal Coordinates Analysis.

High‐throughput sequencing of the V1‐V2 hypervariable region of bacterial 16S rRNA gene was performed by Eurofins Genomics (Eurofins Genomics, Constance, Germany) on an Illumina MiSeq 300 platform. Amplicons were generated using a two‐step PCR protocol with template‐specific primers directed to the V1‐V2 region. Forward PCR primer 5′‐AGAGTTTGATYMTGGCTCAG‐3′ and Reverse PCR primer 5′‐TGCTGCCTCCCGTAGRAGT‐ 3′ were used. A negative control (no template) sample was included on each PCR plate. Cleaning, quantifying and pooling the final amplicon libraries were done at equimolar concentrations. Quantification and sequencing of the resulting library pool was executed using the Illumina MiSeq v3 chemistry (2×300 bp paired end reads) (Figure 1). The sequences are available in the European Nucleotide Archive database under accession number PRJEB66454.

Microbiome analysis and profiling is explained in detail in Data S1.

### Statistical analysis

The alpha and beta diversities were carried out using the *phyloseq* package (version 1.36.0) in R (version 4.1). Alpha diversity was determined using Observed Amplicon Sequence Variants (ASVs) and Shannon indices to measure the richness and diversity, respectively. To compare variation in microbial composition between groups (beta diversity), a Principal Coordinate Analysis (PCoA) was performed based on a Weighted Unifrac distance matrix. Statistical analyses were undertaken using IBM® SPSS® (Version 15.0). Mann–Whitney test was used to compare differences in alpha diversity and in abundance of phyla between the saliva, blood and intracanal samples. The Wilcoxon test was used to compare between group differences in alpha diversity. Linear discriminant analysis Effect Size (LEfSe) was used to compare the biological relevance between symptomatic and asymptomatic intracanal samples. Serum and salivary biomarkers levels analysed in our previous studies (Bakhsh et al., 2022, 2023) were used to investigate correlation between microbial abundance and biomarker levels using Spearman's correlation coefficient. Prior to multiple comparisons, Bonferroni test was used for correction of several dependent and independent tests.

## RESULTS

### Demographic data

Overall, 65 subjects with AP [24 males, 41 females; mean age: 43.3 (24–75)] were recruited. Forty‐five subjects presented with symptoms (four with abscess, 12 with sinus, 29 without abscess/sinus) and 20 were asymptomatic (three with abscess, two with sinus, 15 without abscess/sinus).

### Microbiome analysis

From the DNA extracts sent for sequencing, 169 had positive amplicons (42 extracted DNA from blood, 65 from intracanal and 62 from saliva samples were analysed). The total number of read pairs was 5 906 945. A total of 81.4% (*n* = 5 851 785) read pairs were kept after removal of chimeras. Information on the FASTQ processing results is provided in Table S1.

### Alpha and Beta diversity

**FIGURE 2:**
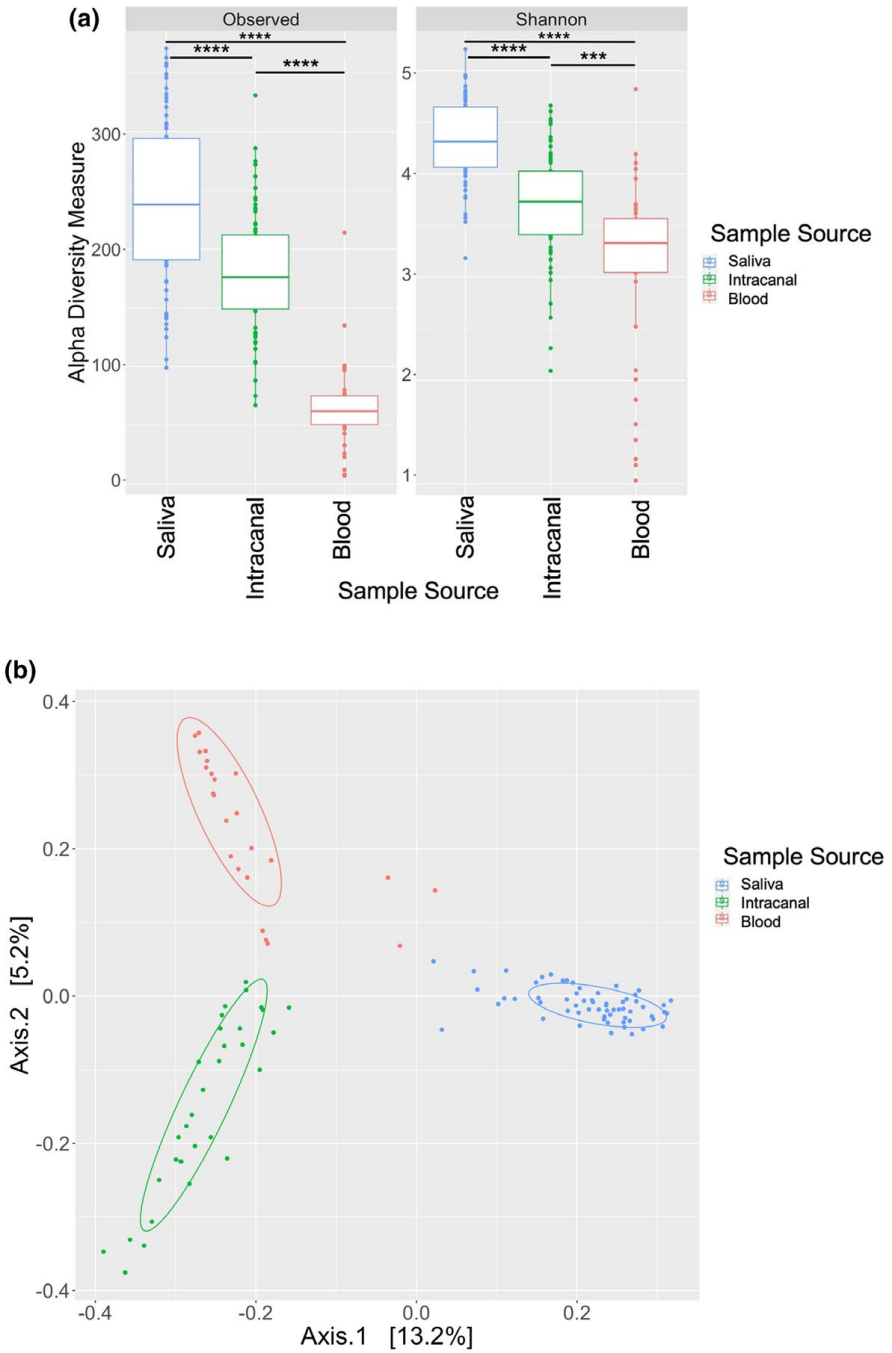
(a) Alpha diversity using Observed and Shannon indices between different sample sources. (b) Beta diversity using Principal Coordinate Analysis (PCoA) plot clustering different sample sources. Samples from the same source are wrapped with an eclipse representing 95% confidence interval.(***p < .001; ****p < .0001)

Alpha diversity of the saliva samples had the highest richness and diversity when analysed using both Observed (mean = 240.7) (*p* < .0001) and Shannon indices (mean = 4.3) (*p* < .0001), followed by intracanal samples (Observed index mean = 179.7 (*p* < .0001); Shannon index mean = 3.7 (*p* < .0001)). Blood samples were the least diverse and rich amongst the different sample sources (Observed index mean = 65.6 (*p* = .0007); Shannon index mean = 3.1 (*p* < .0001)) (Figure 2a). Principal Coordinate Analysis (PCoA) plot with clustering of samples according to the sample source showed minimal similarities between their abundance and diversity (Figure 2b).

### Phyla identified in different sample sources

The comparison of different phyla between sample sources is found in Figure S1.

### Abundant genera found in all sample sources

**FIGURE 3:**
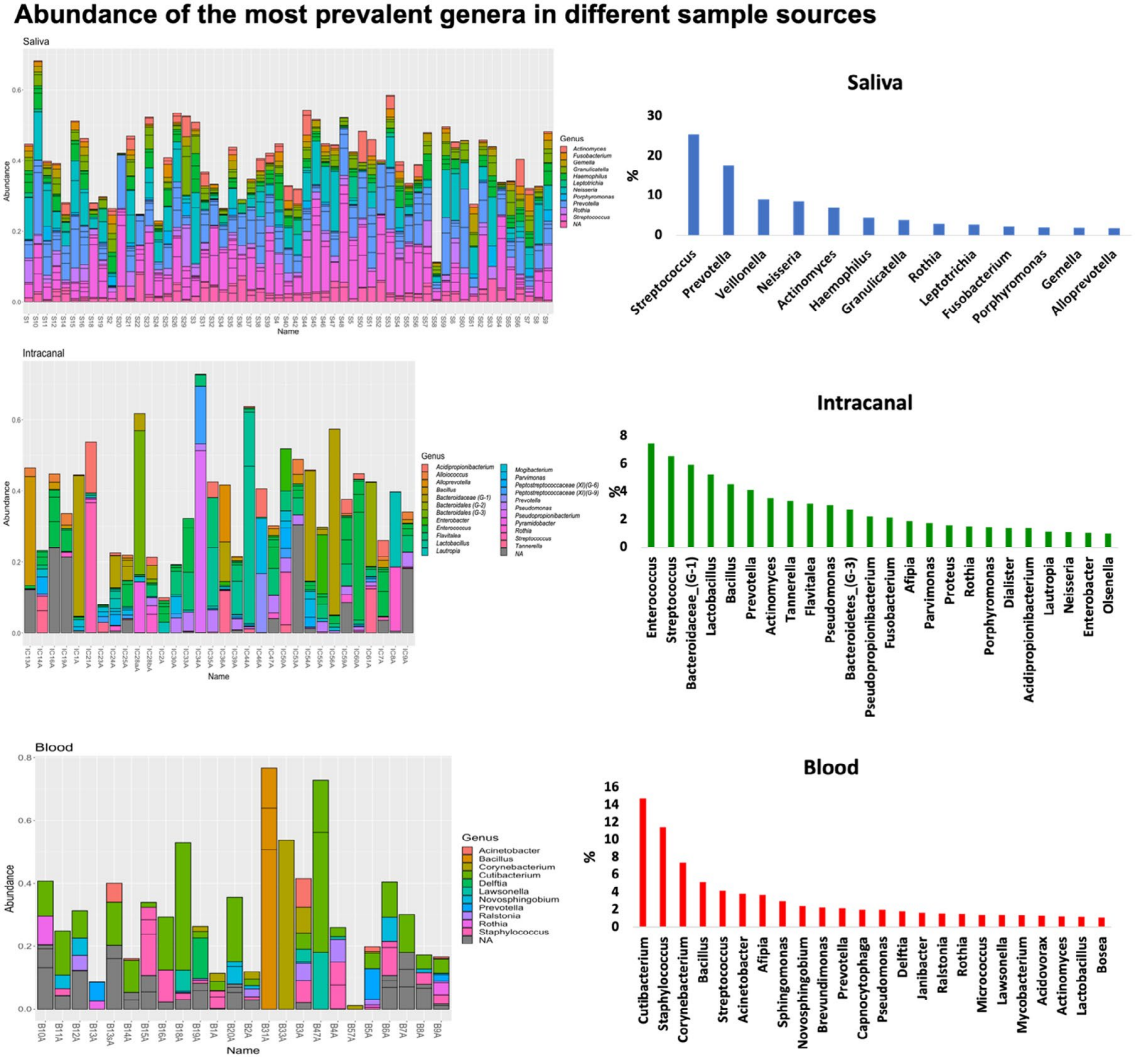
Average abundance of the most prevalent genera in different sample sources (a) identified genera in saliva samples; (b) average abundance of identified genera in saliva samples; (c) identified genera in intracanal samples; (d) average abundance of identified genera in intracanal samples; (e) identified genera in blood samples; (f) average abundance of identified genera in blood samples.

Using the HOMD database, 201 different genera were identified. The most prevalent genera in saliva samples were *Streptococcus and Prevotella*; in intracanal samples were *Enterococcus*, *Streptococcus and Bacteroidaceae* (*G‐1*); and in blood samples were *Cutibacterium* and *Staphylococcus*. However, common taxa were identified amongst all three sample sources with most common ones including *Streptococcus*, *Prevotella*, *Actinomyces* and *Rothia* genera. *Neisseria*, *Fusobacteria* and *Porphyromonas* were common in saliva and intracanal samples, whereas *Lactobacillus*, *Pseudomonas* and *Afipia* were common in blood and intracanal samples (Figure 3a–f).

### Comparison between asymptomatic and symptomatic cases

**FIGURE 4:**
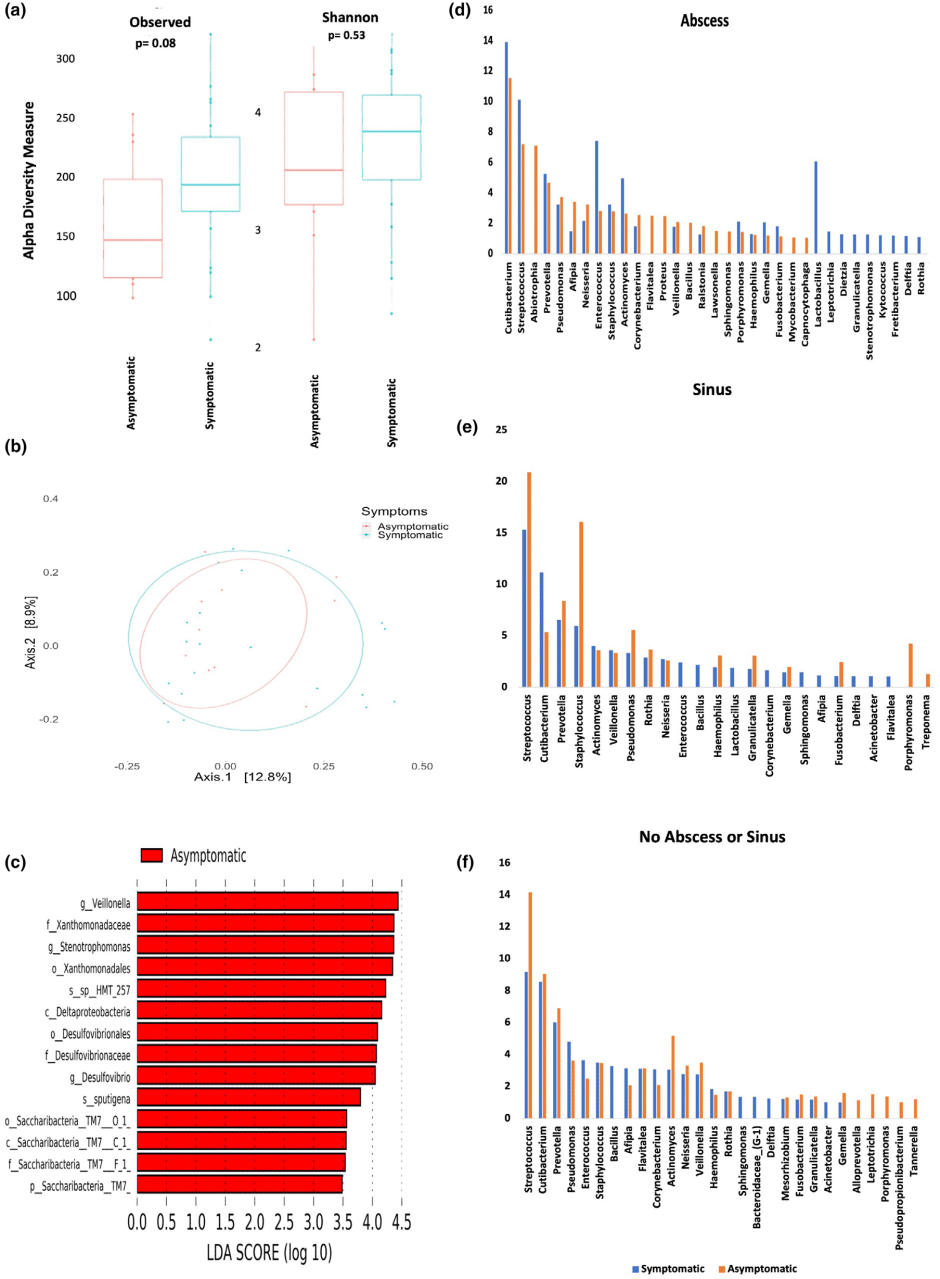
(a) alpha diversity using both observed and Shannon indices between intracanal samples in both asymptomatic and symptomatic cases; (b) beta diversity using PCoA plot between intracanal samples in asymptomatic and symptomatic cases, eclipse showing 95% confidence interval; (c) Linear discriminant analysis Effect Size (LEfSe) between symptomatic and asymptomatic cases showing significantly abundant taxa in asymptomatic cases; (d–f) Average abundance of identified genera in intracanal samples in both symptomatic and asymptomatic cases; (d) showing cases with abscess; (e) showing cases with sinus; (f) showing cases with no abscess or sinus.

Alpha diversity measured by Observed ASVs and Shannon indices for intracanal samples showed higher richness and diversity in symptomatic cases than asymptomatic cases with near significant difference in observed index, but with no significant difference between the means of both groups in the Shannon index (*p* = .08, *p* = .53). Beta diversity showed almost equal distribution for both symptomatic and asymptomatic intracanal samples (Figure 4a,b). Linear discriminant analysis Effect Size (LEfSe) revealed that the *Veillonella*, *Stenotrophomonas* and *Desulfovibrio* genera were higher in abundance in asymptomatic samples (Figure 4c).

Cases with abscess, whether symptomatic or asymptomatic, had more diverse genera than cases with sinus or without abscess/sinus (Figure 4d,e). *Cutibacterium* genus was highly abundant in cases with abscess followed by *Streptococcus*, regardless of symptoms. In cases with sinus, *Streptococcus* were also highly abundant, whether symptomatic or asymptomatic. However, in cases without abscess/sinus, *Enterococcus* was statistically higher in abundance in symptomatic cases (*p* = .02), whilst *Streptococcus* were significantly higher in asymptomatic cases (*p* = .02) (Figure 4d–f).

### Microbial genera in the saliva samples correlation with inflammatory mediators in the saliva

**FIGURE 5:**
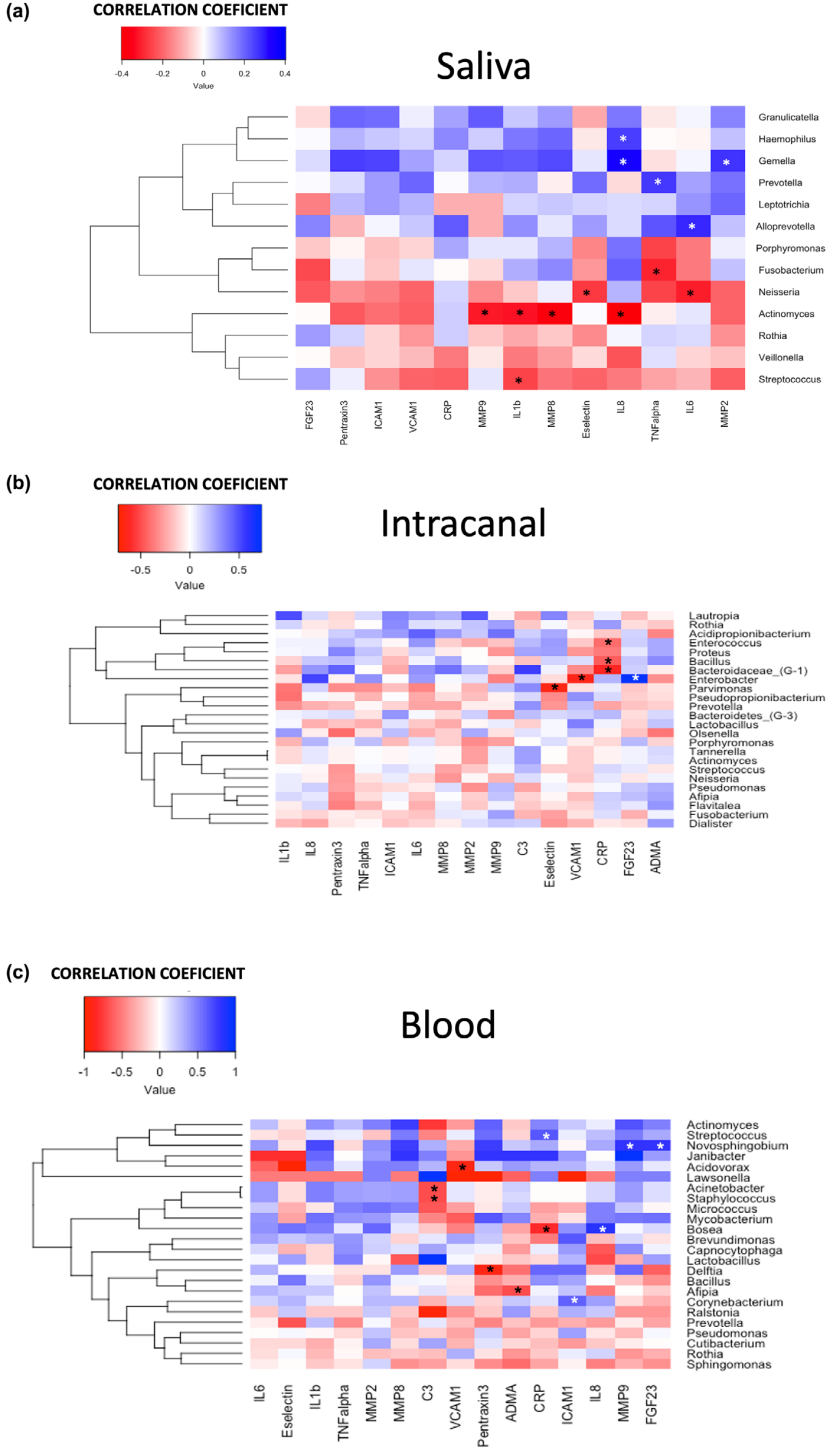
Correlation between abundant genera and the levels of inflammatory markers. (a) correlation between abundant genera in saliva with the levels of salivary inflammatory markers; (b) correlation between abundant genera in intracanal samples with the levels of serum inflammatory markers; (c) Correlation between the abundant genera in blood samples with the levels of serum inflammatory markers. (white *: Positive correlation; black *: Negative correlation).

Correlating salivary microbiome with the previously reported salivary inflammatory markers levels (Bakhsh et al., 2023) revealed significantly positive correlations between the abundance of the *Haemophilus* with IL‐8 levels (*r* = .25, *p* = .049), *Gemella* with MMP‐2 and IL‐8 (*r* = .27, *p* = .04; *r* = .35, *p* = .007 respectively), *Prevotella* with TNF‐α (*r* = .25, *p* = .044) and *Alloprevotella* with IL‐6 levels (*r* = .28, *p* = .026) (Figure 5a).

In contrast, negative correlation was found between abundance of the *Actinomyces* with levels of MMP‐8, MMP‐9, IL‐1β and IL‐8 (*r* = −.38, *p* = .002; *r* = −.31, *p* = .013; *r* = −.32, *p* = .011; and *r* = −.40, *p* = .001, respectively). A significant negative correlation was also evident between *Fusobacterium* and TNF‐α (*r* = −.30, *p* = .019); *Neisseria* and IL‐6 with E‐selectin (*r* = −.31, *p* = .017; *r* = −.27, *p* = .036 respectively); and *Streptococcus* with IL‐1β (*p* = .045).

### Microbial genera in the intracanal samples correlation with the inflammatory mediators in serum

The abundance of the *Enterobacter* in intracanal samples was positively correlated with serum levels of FGF‐23 (*r* = .72, *p* = .007) and negatively correlated with VCAM‐1 (*r* = −.64, *p* = .024). *Bacteroidetes (G‐1)* was negatively correlated with CRP levels (*r* = −.61, *p* = .044). *Enterococcus* and *Bacillus* were negatively correlated with CRP levels (*r* = −.41, *p* = .027; *r* = −.46 *p* = .048, respectively) (Figure 5b), whilst the *Parvinomonas* was negatively correlated with E‐selectin levels (*r* = −.66, *p* = .001).

### Microbial genera in the blood samples correlation with the inflammatory mediators in serum

Blood bacteraemia was significantly correlated with the serum inflammatory biomarkers reported in Bakhsh et al. (2022). The *Novosphingobium* was positively correlated with FGF‐23 and MMP‐9 (*r* = .78, *p* = .036; *r* = .78, *p* = .036, respectively). The *Streptococcus*, *Bosea* and *Corynebacterium* genera were positively correlated with CRP, IL‐8, and ICAM‐1, respectively (*r* = .30, *p* = .043; *r* = .82, *p* = .042; *r* = .61, *p* = .025). However, *Bosea* was negatively correlated with the CRP (*r* = −.82, *p* = .042). Both *Staphylococcus* and *Actinobacter* were negatively correlated with C3 (*r* = −.69, *p* = .019; *r* = −.69, *p* = .019 respectively), whilst *Acidovorax* were negatively correlated with VCAM‐1 (*r* = −.90, *p* = .037). Moreover, *Delftia* was negatively correlated with pentraxin 3 (*r* = −.90, *p* = .037; and *Afipia* was negatively correlated with ADMA (*p* = .021, rho = −.68)) (Figure 5c).

## DISCUSSION

Ingress of bacteria into the root canal system causes pulp necrosis and subsequent AP. This study revealed that specific intracanal and blood bacterial genera are associated with increase of serum CVDs risk biomarkers such as FGF‐23 and hs‐CRP (Niazi & Bakhsh, 2022). We have shown in our previous study that successful endodontic treatment led to a significant reduction in these markers at 1‐ and 2‐year post‐treatment follow‐ups (Al‐Abdulla et al., 2023; Bakhsh et al., 2022).

Recent advances in microbiome detection challenge the notion of sterile blood (Castillo et al., 2019), although this is still a highly debated theory. This study aligns with other research suggesting that human blood may contain microbiota, albeit at lower richness and diversity when compared to other sample sources. Even though Next‐generation sequencing is a sensitive molecular approach in detecting bacteria when compared to culture techniques, one of its limitations is that sequencing of low‐quality and/or ‐quantity bacterial DNA is challenging. However, this next‐generation sequencing approach using targeted 16SrRNA gene sequencing analysis had been successfully used previously to investigate blood microbiome (Qian et al., 2018). Previous studies found that the most abundant phyla in blood are from skin and oral communities including *Proteobacteria*, *Actinobacteria*, *Firmicutes* and *Bacteroidetes* (Whittle et al., 2019; Alquria et al., 2024a). Our data also suggest that microbes belonging to these phyla could disseminate from the root canal into systemic circulation causing low‐grade systemic inflammation and other effects. However, one of the limitations of our study was absence of a control group, which could further strengthen the evidence regarding bacteraemia, and this is an area which can be investigated in future studies.

Amongst the analysed sources, saliva had the highest diversity likely due to the environmental factors, dietary habits, oral disease and systemic condition of the patient. The most abundant salivary genera included *Veillonella, Actinomyces, Granulicatella, Leptotrichia, Fusobacterium, Gemella* and *Alloprevotella*, which are pathobionts in the oral cavity, intestine and skin (Li et al., 2020; Zhou & Li, 2021). However, they have also been linked to several dental and systemic conditions including periodontitis, AP, chronic obstructive disease and infective endocarditis (Persoon et al., 2017).

The composition of intracanal samples is influenced by factors such as detection technique sensitivity, geographical location of the study, stage of disease progression and clinical presentation (Siqueira Jr. & Rôças, 2022). In this study, *Enterococcus* were most abundant in intracanal samples recovered from these retreatment cases, which is consistent with other studies associating it with secondary/persistent root canal infection due to their resilience in harsh conditions (Siqueira Jr. et al., 2016). *Streptococcus* genus was also abundant, similar to previous studies on treated root canals, due to their ubiquity in the oral cavity (Zandi et al., 2018). Furthermore, *Lactobacillus, Prevotella and Tannerella* were also amongst the abundant intracanal genera. *Lactobacillus* is one of the important bacteria involved in caries (Wen et al., 2022), whereas, *Prevotella* and *Tannerella* are major periodontal pathogens (Jung et al., 2021), suggesting that caries and supra‐ or sub‐gingival plaque may contribute microbes to the root canal.

In this study, symptomatic AP subjects exhibited higher microbiota richness and diversity compared to asymptomatic subjects. Results showed relatively high abundance of the phylum *Saccharibacteria* (TM7) and genera *Veillonella* in asymptomatic cases. *Saccharibacteria* (TM7) are oral pathogens, associated with oral‐mucosal infection and secondary cardiovascular events (Schulz et al., 2021). *Veillonella* spp. include oral pathogens which are influenced by the *Streptococcal* species biofilm formed (Mashima & Nakazawa, 2014). Rôças and Siqueira Jr. (2018) found that both symptomatic and asymptomatic cases were associated with *Streptococcus, Treponema* and *Porphyromonas* genera, although they were more abundant in cases with abscess (Rôças & Siqueira Jr., 2018). In our study, *Cutibacterium* and *Streptococcus* genera were abundant in cases with abscess. In the study by Niazi et al. (2010, 2016), *Cutibacterium acnes*—a known opportunistic pathogen, responsible for inflammatory conditions and nosocomial infections (Günthard et al., 1994)—were isolated from refractory endodontic lesions with or without abscesses and primary endodontic lesions with oral communication (Niazi et al., 2010; Niazi et al., 2016; Alquria et al., 2024b).

Bacteria and their by‐products cause up‐regulation of several local and systemic inflammatory markers which may lead to systemic complications (Martinho et al., 2021). This study highlights the systemic host–microbiome interactions by identifying correlations between the levels of serum inflammatory markers and the abundance of specific bacterial species in intracanal, and blood samples, along with the local host–microbiome interactions through correlation between levels of salivary inflammatory markers with the abundance of specific salivary microbiome. Interestingly, these correlations were not specific to Gram‐negative species, indicating that interactions were more than just a response to shed lipopolysaccharide (LPS). In saliva, levels of IL‐8 positively correlated with *Haemophilus* spp. (*p* = .049), a Gram‐negative taxon associated with chronic obstructive pulmonary disease (Wang et al., 2003). Wang et al. (2003) found that *Haemophilus* induced the production of IL‐8 through the activation of extracellular signal‐regulated kinase mitogen‐activated protein kinase (ERK–MAPK) pathway. Furthermore, *Gemella* spp. were positively correlated with the salivary levels of MMP‐2. *Gemella* are Gram‐positive bacteria which can increase the production of MMP‐2 during AP progression (Martinho et al., 2016; Takeda & Akira, 2005). *Prevotella* spp. were positively correlated with the salivary TNF‐α level (*p* = 0.044). The results are in line with the results of Kim et al. (2007) who found that the LPS of *Prevotella* stimulates the release of TNF‐α through the MAPK signalling pathway in monocyte‐derived macrophages. In addition, *Alloprevotella* was positively correlated with the salivary levels of IL‐6. *Alloprevotella* which is a Gram‐negative anaerobic genus are commonly isolated from the oral cavity and are usually associated with oral infections. Notably, it has been found that Gram‐negative bacteria induce higher levels of IL‐6 than Gram‐positive bacteria (Kragsbjerg et al., 1998).

The effect of the intracanal microbiome on the serum inflammatory markers levels showed a positive correlation between *Enterobacter* and the metabolism modifier, FGF‐23. Other than its role in inflammation, it had also been identified as novel markers for CVD risk (Niazi & Bakhsh, 2022). FGF‐23, a bone‐derived hormone, is produced by osteoblasts and osteocytes (Quarles, 2012). The serum levels of FGF‐23 are upregulated by inflammation, infection and oxidative stress (Ito et al., 2015). Thus, increased FGF‐23 levels in our study reflected increased AP systemic burden. Notably, in mice, Masuda et al. (2015) found that Gram‐negative bacteria increase in the serum FGF‐23 levels, which is in line with our findings.

There were correlations between serum levels of the inflammatory markers FGF‐23 and MMP‐9 and the abundance of *Novosphingobium* in blood, along with a positive correlation between the levels of hs‐CRP, IL‐8 and ICAM‐1 and the abundance of *Streptococcus, Bosea* and *Corynebacterium*, respectively. In all cases, presence of these microbial species is associated with a general increase in inflammation. *Novosphingobium*, known to activate natural killer T‐cells, which cause an increase in MMP‐9 levels, explains the positive correlation between the MMP‐9 serum levels and *Novosphingobium* abundance in blood (Mak & Saunders, 2013). The *Streptococcus* in blood was positively correlated with the serum hs‐CRP levels, in line with studies assessing the same correlation in subjects with streptococcal tonsillitis (Koo & Eisenhut, 2011). Although *Streptococcus* being primary colonizers in plaque, have some species identified as dental caries pathogens, and others associated with bacteraemia and infective endocarditis (Nakano et al., 2008). Furthermore, Gram‐positive *Corynebacterium in blood* were positively correlated with the ICAM‐1 serum levels. This tallies with a previous study that found ICAM‐1 levels were up‐regulated in mice in the presence of *Corynebacterium* (Steffen et al., 1996). To our knowledge, this is the first study demonstrating how the microbiome of saliva, intracanal and blood of subjects with AP can influence the levels of some of the inflammatory markers in saliva and serum, thus impacting local and systemic health.

## CONCLUSION

The diversity and richness of the genera in symptomatic cases were higher compared to asymptomatic ones. Salivary, tooth‐intracanal and blood microbiome of AP subjects showed common microbial taxa suggesting root canal infections containing salivary pathogens are the potential source of bacteraemia associated with AP. Microbiome in saliva, blood and intracanal samples were associated with some of the salivary and serum inflammatory biomarker levels, highlighting the local and systemic host–microbiome interactions in AP. This suggests that the effect of AP goes beyond a periapical infection, as systemic inflammatory burden caused by AP may potentiate risk for systemic conditions if left untreated.

## AUTHOR CONTRIBUTIONS

**Bakhsh A:** Conceptualization; data curation; formal analysis; investigation; methodology; resources; project administration; validation; visualization; writing—original draft; writing—review & editing. **Joseph S:** Formal analysis, visualization, writing—original draft; writing— review and editing. **Mannocci F:** Supervision; writing—review and editing. **Proctor G:** Resources; writing—review and editing. **Moyes D:** Resources; supervision; writing—review and editing. **Niazi S:** Conceptualization; data curation; funding acquisition; resources, supervision, methodology; project administration; validation; visualization; writing—original draft; writing—review and editing.

## FUNDING INFORMATION

Funding was received by the British Endodontic Society (Grant for Research Work) and European Society of Endodontology (Annual Research Grant). Sadia Ambreen Niazi is the principal grant holder of these grants.

## CONFLICT OF INTEREST STATEMENT

The authors deny any conflicts of interest related to this study.

## ETHICS STATEMENT

The protocol was approved by the London‐Hampstead Research Ethics Committee (IRAS project ID 207795). Written consent was obtained in accordance with the Declaration of Helsinki.

## Supporting information


Data S1:

## Data Availability

The data that support the findings of this study are openly available in European Nucleotide Archive database at https://www.ebi.ac.uk/ena/browser/view/PRJEB66454, reference number PRJEB66454.
