# Supplementary material for: Apical periodontitis microbiome association with salivary and serum inflammatory burden
Source: Int Endod J. 2025 Jan 4;58(3):504–15. doi: 10.1111/iej.14184 (PMC11812624; doi:10.1111/iej.14184)
Supplement: Supplementary file 1 — Data S1: [file IEJ-58-504-s001.docx]

**Apical periodontitis microbiome association with salivary and serum inflammatory burden**

**Sampling Protocol**

***Saliva sample Collection***

Unstimulated pre-operative saliva samples were collected from all three groups in a polystyrene 30 ml universal tube (Fisher Scientific, Waltham, MA, USA) for 10 min. The collected saliva was transported on ice and stored in a -80 °C freezer (Sanyo Electric Co, Japan).

***Blood sample collection***

Blood samples from the control group were collected using BD Vacutainer SST II Advance 8.5 ml tube (Becton Dickinson, NJ, USA). In contrast, for both treatment groups, pre- and post-operative blood samples were collected using a 4 ml Vacuette K3 EDTA blood tube (Greiner Bio-One, Austria) and BD Vacutainer SST II Advance 8.5 ml tube (Becton Dickinson). For bacterial culture, blood was also withdrawn into BD Bactec ANO2 10 ml bottle (Becton Dickinson).

Whole blood collected in the 4 ml Vacuette K3 EDTA tube (Greiner Bio-One) was then separated into aliquots of 0.5 ml in a 1.5 ml microcentrifuge tube (Fisher Scientific) then stored in a -80 °C freezer. Blood samples collected in the BD Vacutainer SST II Advance 8.5 ml tube (Becton Dickinson) from all three groups were centrifuged for 10 min at a speed of 2000 x g and a temperature of 4 °C using Eppendorf centrifuge 5810R (Eppendorf, Hamburg, Germany) to separate the serum from the blood. The separated serum was then stored in a -80 °C freezer in an aliquot of 0.5 ml in a 1.5 ml microcentrifuge tube (Fisher Scientific) for further processing.

***Root canal sample collection***

A single, trained operator collected all the samples under aseptic conditions. Local anaesthesia was administered, and each target tooth was cleaned with pumice. This was followed by rubber dam isolation (UnoDent, UK) with an appropriate clamp and sealed with a light-cured gingival barrier, OpalDam Green™ (Ultradent Products, Inc., South Jordon, UT, USA). The tooth surface was disinfected using a small cotton pellet immersed in 2.5% sodium hypochlorite (NaOCl) (Milton Laboratories, Rivadis, Louzy, France), followed by 5% sodium thiosulfate swabbed for at least 30 s. A contamination control sample was taken using sterile swab rubbed on the tooth surface for 30 s (These were check later by culture and DNA extraction. All these control samples showed negative results).

A sterile bur cooled with saline delivered with a separate syringe was used to remove all restorations and caries. The rubber dam and tooth were again disinfected with 2.5% NaOCl (Milton Laboratories). The tooth was then built up using composite restoration (SDR flow+; Dentsply Sirona, Baillagues, Switzerland) to avoid leakage. Access was gained with a new sterile bur and saline (Sterets Normasol, Oldham, UK) exposing the pulp chamber. Single-use sterile Gates Glidden burs, Hedstrom files (H-files), Flexofiles (Dentsply Sirona), and rotary files were used to remove existing root canal filling with the use of sterile saline (Sterets Normasol). Instruments were separated from their heads using a sterile bur and were placed into a 7 ml bijou tube (Greiner Bio-One Ltd) along with the root canal filling material; the bijou tube also contained 1 ml of fastidious anaerobic broth (Lab M). Canals were filled with saline (Sterets Normasol), and paper points (Dentsply Sirona) samples were collected. Samples were transported in ice and stored in a -80 °C freezer for further processing.

**Phyla identified in different sample sources**

Using the HOMD database, 201 different genera belonging to 12 distinct phyla including *Firmicutes*, *Actinobacteria*, *Proteobacteria*, *Bacteroidetes*, *Fusobacteria*, *Saccharibacteria (TM7), Spitochaetes, Synergistetes, Absconditabacteria (SR1), Gracilibacteria (GN02), Chloroflexi* and *Cyanobacteria* were identified from all three sample sources. *Actinobacteria* were significantly higher in abundance than in blood samples than in saliva samples and intracanal samples (p<0.0001, p<0.0001, respectively), while a significant difference was also present between saliva samples and intracanal samples (p<0.0001). In contrast, *Bacteroidetes* were significantly higher in saliva samples and intracanal samples than in blood samples (p<0.0001). A significant difference was also found in *Firmicutes* between saliva samples and blood and intracanal samples (p<0.0001, p=0.03, respectively). *Firmicutes* were also significantly higher in intracanal samples than in blood samples (p=0.01). Abundance of the *Fusobacteria* phylum was found at significantly higher levels in saliva samples than in blood and intracanal samples (p=0.007, p<0.0001, respectively), whilst *Proteobacteria* were significantly higher in blood than in intracanal and saliva samples (p=0.002, p<0.0001) and higher in intracanal than in saliva samples (p=0.0006). *Saccharibacteria (TM7)* was significantly higher in blood than in intracanal samples and significantly higher in saliva samples than in intracanal samples (p=0.0004 and p<0.0001, respectively). Moreover, *Spirochaetes* was significantly lower in saliva samples than in blood and intracanal samples (p=0.006 and p=0.0004), while *Synergistetes* was significantly higher in intracanal samples than in saliva samples (p<0.0001).

### **Microbiome analysis**

All reads passing the standard Illumina filter procedure (chastity filter) were demultiplexed and corresponded with the sequence of their sample. The region-specific forward and reverse primer sequence were identified and clipped from the start of raw forward and reverse reads. Unmatched read pairs were removed to maintain high-quality ones. The forward and reverse clipped FASTQ reads were provided by Eurofins for each sample. The FASTQ files were processed using *DADA2* (version 1.2) in R (version 4.1). After importing the FASTQ files, the quality profile of each forward and reverse was obtained by plotting the average quality score. The low-quality sequences were filtered, trimmed, and truncated. Truncation was set to 200bp for the forward files and 175bp for the reverse files. Error frequency was checked. Identical sequence reads were dereplicated into a single unique sequence with a corresponding abundance. Forward and reverse reads were merged to generate amplicon sequence variants (ASVs). Chimeric reads were identified and removed. The counts were normalised using the median of ratios method in DeSeq2 (version 1.32.0) package in R. The information on the FASTQ Read statistics is provided in supplementary Table 1.

### **Microbiome profiling**

The merged and clipped pair reads were used as input for microbiome profiling. Each sequence variant was classified by comparison with a specific database using the Naïve Bayesian Classifier Method. The Human Oral Microbiome Database (HOMD) (version 15.1) was used for assigning taxonomy after generating the phyloseq object using the *phyloseq* package (version 1.36.0) and plots were generated using *ggplot2* (version 3.35) for the generated ASVs.


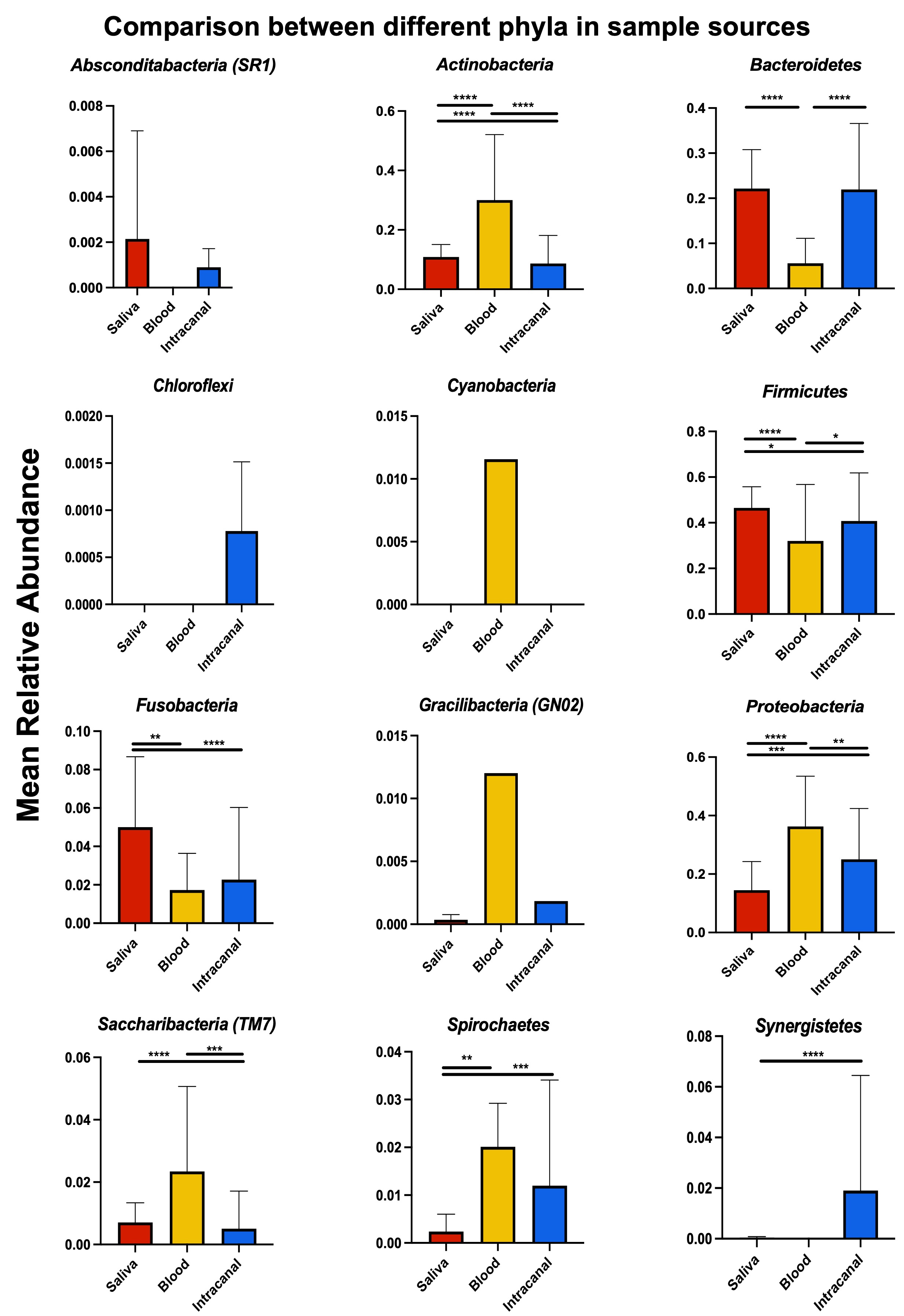


**Supplementary figure 1:** Comparison between different phyla in sample sources

**Supplementary Table 1**: FASTQ processing results.

|  | **Read Pairs** | **Filtered** | **Denoised Forward** | **Denoised Reverse** | **Merged** | **Nonchimera** | **Percentage kept** |
| --- | --- | --- | --- | --- | --- | --- | --- |
| 1 | 39054 | 33182 | 33015 | 32991 | 32502 | 32195 | 82.43713832 |
| 2 | 39992 | 34371 | 34135 | 34218 | 33519 | 33467 | 83.68423685 |
| 3 | 29778 | 25030 | 24773 | 24709 | 23954 | 23909 | 80.29081873 |
| 4 | 18347 | 15661 | 15418 | 15491 | 14984 | 14982 | 81.65912683 |
| 5 | 44231 | 37499 | 37330 | 37319 | 36483 | 36477 | 82.46930886 |
| 6 | 37737 | 31488 | 31297 | 31270 | 30557 | 30451 | 80.69268887 |
| 7 | 33918 | 28753 | 28497 | 28528 | 27223 | 27189 | 80.16097647 |
| 8 | 32963 | 28022 | 27839 | 27863 | 27380 | 27213 | 82.55619938 |
| 9 | 33970 | 28201 | 28122 | 28133 | 27925 | 27842 | 81.96055343 |
| 10 | 24099 | 20585 | 20347 | 20384 | 19599 | 19567 | 81.19424042 |
| 11 | 41614 | 35574 | 35236 | 35225 | 34191 | 34057 | 81.84024607 |
| 12 | 21901 | 18441 | 18203 | 18194 | 17852 | 17791 | 81.23373362 |
| 13 | 44242 | 37418 | 37238 | 37223 | 36462 | 36171 | 81.75715384 |
| 14 | 29759 | 25285 | 25066 | 25027 | 24346 | 24341 | 81.79374307 |
| 15 | 37289 | 31898 | 31664 | 31761 | 30996 | 30886 | 82.82871624 |
| 16 | 37398 | 32090 | 31607 | 31735 | 30068 | 30036 | 80.31445532 |
| 17 | 38662 | 32856 | 32624 | 32689 | 31854 | 31842 | 82.35993999 |
| 18 | 34877 | 28327 | 28215 | 27632 | 27462 | 27385 | 78.51879462 |
| 19 | 40692 | 34796 | 34471 | 34700 | 34023 | 33995 | 83.5422196 |
| 20 | 32495 | 27291 | 27092 | 27102 | 26361 | 26345 | 81.07401139 |
| 21 | 33588 | 28541 | 28380 | 28446 | 28111 | 28024 | 83.43455996 |
| 22 | 33067 | 27915 | 27817 | 27770 | 27358 | 27253 | 82.41751595 |
| 23 | 32744 | 27897 | 27760 | 27820 | 27552 | 27412 | 83.71610066 |
| 24 | 39030 | 33164 | 32982 | 32941 | 32295 | 32043 | 82.09838586 |
| 25 | 34969 | 29923 | 29687 | 29737 | 29099 | 28947 | 82.77903286 |
| 26 | 35310 | 29960 | 29751 | 29757 | 28861 | 28851 | 81.70773152 |
| 27 | 25537 | 21511 | 21328 | 21388 | 20741 | 20607 | 80.69467831 |
| 28 | 40509 | 33894 | 33762 | 33754 | 33285 | 33266 | 82.12002271 |
| 29 | 28872 | 24743 | 24527 | 24564 | 23878 | 23873 | 82.68564699 |
| 30 | 43784 | 37253 | 37112 | 37172 | 36424 | 36403 | 83.14224374 |
| 31 | 41618 | 36114 | 35758 | 35880 | 35054 | 35033 | 84.17751934 |
| 32 | 28964 | 24284 | 24046 | 24052 | 23393 | 23387 | 80.74506284 |
| 33 | 38743 | 33595 | 33325 | 33474 | 32922 | 32876 | 84.85661926 |
| 34 | 41421 | 35156 | 34927 | 35013 | 34008 | 33724 | 81.4176384 |
| 35 | 31138 | 26483 | 26257 | 26291 | 25717 | 25641 | 82.34632924 |
| 36 | 39096 | 33021 | 32819 | 32816 | 32342 | 31909 | 81.61704522 |
| 37 | 39665 | 33145 | 32939 | 32962 | 32274 | 32251 | 81.30845834 |
| 38 | 31389 | 26661 | 26526 | 26500 | 26109 | 26099 | 83.14696231 |
| 39 | 40790 | 34666 | 34415 | 34467 | 33465 | 33437 | 81.97352292 |
| 40 | 39228 | 32990 | 32629 | 32737 | 31385 | 31229 | 79.60895279 |
| 41 | 12118 | 10348 | 10248 | 10243 | 9883 | 9839 | 81.19326622 |
| 42 | 20885 | 17795 | 17656 | 17632 | 17201 | 17184 | 82.27914771 |
| 43 | 40240 | 34165 | 33850 | 33903 | 33145 | 33064 | 82.16699801 |
| 44 | 42461 | 36003 | 35759 | 35821 | 34956 | 34613 | 81.51715692 |
| 45 | 48919 | 41794 | 41561 | 41558 | 40704 | 40532 | 82.85533228 |
| 46 | 34908 | 29277 | 28903 | 29025 | 28399 | 28389 | 81.32519766 |
| 47 | 41832 | 35702 | 35275 | 35440 | 34037 | 33852 | 80.92369478 |
| 48 | 32797 | 27832 | 27617 | 27671 | 27167 | 27113 | 82.66914657 |
| 49 | 33215 | 28411 | 28125 | 28202 | 27608 | 27558 | 82.96853831 |
| 50 | 26669 | 22614 | 22395 | 22397 | 22164 | 22157 | 83.08148037 |
| 51 | 26166 | 22336 | 22139 | 22201 | 21619 | 21611 | 82.59191317 |
| 52 | 43725 | 36948 | 36647 | 36727 | 36093 | 36053 | 82.4539737 |
| 53 | 36752 | 30929 | 30779 | 30701 | 30284 | 30281 | 82.39279495 |
| 54 | 32465 | 26994 | 26714 | 26726 | 26084 | 26054 | 80.2525797 |
| 55 | 40444 | 34359 | 34209 | 34153 | 33419 | 33408 | 82.60310553 |
| 56 | 36414 | 30585 | 30343 | 30427 | 29779 | 29637 | 81.3890262 |
| 57 | 33034 | 28083 | 27921 | 27902 | 27278 | 27256 | 82.50893019 |
| 58 | 35140 | 30169 | 29968 | 29955 | 29468 | 28865 | 82.14285714 |
| 59 | 44022 | 37057 | 36854 | 36874 | 36235 | 36172 | 82.16800691 |
| 60 | 37032 | 30662 | 30345 | 30338 | 29046 | 29007 | 78.32955282 |
| 61 | 42844 | 36051 | 35834 | 35886 | 35232 | 35129 | 81.99281113 |
| 62 | 36283 | 30802 | 30649 | 30624 | 29589 | 29378 | 80.96904887 |
| 63 | 41616 | 34987 | 34782 | 34775 | 34163 | 33936 | 81.5455594 |
| 64 | 37754 | 32150 | 31954 | 31920 | 31034 | 30993 | 82.09196377 |
| 65 | 42482 | 36057 | 35804 | 35914 | 35188 | 35118 | 82.66559955 |
| 66 | 42122 | 35598 | 35519 | 35573 | 34811 | 34684 | 82.34176915 |
| 67 | 45777 | 38872 | 38741 | 38762 | 38028 | 37332 | 81.55187103 |
| 68 | 53396 | 45038 | 44873 | 44889 | 44337 | 44170 | 82.72155218 |
| 69 | 47456 | 40449 | 40258 | 40374 | 39610 | 39284 | 82.77983817 |
| 70 | 46322 | 39051 | 38932 | 38805 | 37906 | 37791 | 81.58326497 |
| 71 | 50374 | 42968 | 42907 | 42918 | 42495 | 41901 | 83.17981498 |
| 72 | 41870 | 35636 | 35550 | 35566 | 35235 | 34935 | 83.43682828 |
| 73 | 42610 | 35940 | 35795 | 35798 | 34605 | 33775 | 79.26543065 |
| 74 | 39117 | 33401 | 33177 | 33317 | 30735 | 30696 | 78.47227548 |
| 75 | 44374 | 38220 | 38132 | 38107 | 37749 | 37663 | 84.8762789 |
| 76 | 42894 | 34303 | 34244 | 34272 | 34215 | 34185 | 79.69646104 |
| 77 | 51738 | 44421 | 44200 | 44311 | 43730 | 43140 | 83.38165372 |
| 78 | 51485 | 43338 | 43263 | 43192 | 42785 | 42665 | 82.86879674 |
| 79 | 51980 | 43342 | 43236 | 43149 | 42119 | 41682 | 80.18853405 |
| 80 | 47796 | 40389 | 40226 | 40336 | 39887 | 38114 | 79.74307473 |
| 81 | 47302 | 39766 | 39595 | 39682 | 39240 | 38889 | 82.21428269 |
| 82 | 57160 | 47844 | 47662 | 47660 | 46647 | 46525 | 81.3943317 |
| 83 | 43521 | 36960 | 36876 | 36764 | 35972 | 35908 | 82.50729533 |
| 84 | 52701 | 44508 | 44358 | 44339 | 43786 | 43249 | 82.06485645 |
| 85 | 46419 | 37287 | 37213 | 37228 | 36152 | 36104 | 77.77849587 |
| 86 | 47755 | 39811 | 39624 | 39609 | 39209 | 38982 | 81.62914878 |
| 87 | 44435 | 37609 | 37476 | 37481 | 36939 | 36377 | 81.86564645 |
| 88 | 53609 | 46029 | 45851 | 45967 | 45406 | 40348 | 75.26348188 |
| 89 | 46655 | 39065 | 39059 | 39059 | 38805 | 37984 | 81.41463937 |
| 90 | 42234 | 35469 | 35448 | 35445 | 34997 | 32164 | 76.15665104 |
| 91 | 47314 | 40621 | 40604 | 40571 | 40401 | 40027 | 84.59863888 |
| 92 | 34809 | 29937 | 29917 | 29920 | 29777 | 28278 | 81.23761096 |
| 93 | 53005 | 44933 | 44695 | 44740 | 43989 | 42310 | 79.82265824 |
| 94 | 49998 | 43293 | 43279 | 43272 | 43067 | 43067 | 86.1374455 |
| 95 | 48460 | 40998 | 40837 | 40965 | 40714 | 40543 | 83.66281469 |
| 96 | 42356 | 35641 | 35530 | 35538 | 35016 | 34899 | 82.39446596 |
| 97 | 41337 | 35393 | 35313 | 35291 | 34853 | 34797 | 84.17882285 |
| 98 | 45389 | 38043 | 38002 | 37964 | 37743 | 37737 | 83.14128974 |
| 99 | 45778 | 38557 | 38371 | 38265 | 37801 | 37414 | 81.72921491 |
| 100 | 43215 | 36967 | 36879 | 36862 | 36037 | 35878 | 83.02209881 |
| 101 | 38827 | 32962 | 32949 | 32859 | 32448 | 32424 | 83.50889845 |
| 102 | 47332 | 39983 | 39780 | 39811 | 39178 | 37841 | 79.9480267 |
| 103 | 45806 | 38587 | 38515 | 38510 | 37931 | 37881 | 82.69877309 |
| 104 | 44365 | 37928 | 37849 | 37798 | 37607 | 37411 | 84.3254818 |
| 105 | 44277 | 37925 | 37857 | 37815 | 37446 | 37412 | 84.49533618 |
| 106 | 42647 | 36046 | 35900 | 35864 | 35551 | 35386 | 82.97418341 |
| 107 | 44755 | 38500 | 38336 | 38352 | 37476 | 37203 | 83.12590772 |
| 108 | 49261 | 42303 | 41908 | 42028 | 40555 | 40420 | 82.05273949 |
| 109 | 55459 | 47539 | 47244 | 47313 | 46653 | 46488 | 83.82408626 |
| 110 | 45861 | 39073 | 38664 | 38760 | 36940 | 36594 | 79.79328841 |
| 111 | 49165 | 41918 | 41646 | 41626 | 39869 | 39174 | 79.67863317 |
| 112 | 30839 | 26396 | 26155 | 26240 | 25488 | 24490 | 79.41243231 |
| 113 | 35780 | 30437 | 30197 | 30106 | 29001 | 28702 | 80.21799888 |
| 114 | 37063 | 32102 | 31911 | 31840 | 30648 | 30475 | 82.22486037 |
| 115 | 44042 | 37986 | 37634 | 37691 | 35654 | 35545 | 80.70705236 |
| 116 | 48048 | 41553 | 41300 | 41368 | 40311 | 40106 | 83.47069597 |
| 117 | 61434 | 52414 | 51959 | 52075 | 50269 | 49926 | 81.26770192 |
| 118 | 60688 | 51846 | 51528 | 51425 | 49900 | 49632 | 81.78223042 |
| 119 | 64524 | 55017 | 54855 | 54773 | 54061 | 52793 | 81.81916806 |
| 120 | 45265 | 38730 | 38364 | 38381 | 36745 | 36080 | 79.70838396 |
| 121 | 44661 | 38572 | 38239 | 38327 | 37387 | 37209 | 83.31430107 |
| 122 | 52056 | 43961 | 43611 | 43713 | 42016 | 41308 | 79.35300446 |
| 123 | 60761 | 52135 | 51588 | 51810 | 49641 | 49268 | 81.08490644 |
| 124 | 37465 | 32172 | 31780 | 31828 | 30619 | 29160 | 77.8326438 |
| 125 | 41722 | 35805 | 35544 | 35580 | 34136 | 32383 | 77.61612578 |
| 126 | 59175 | 50462 | 50093 | 50123 | 47662 | 47149 | 79.67722856 |
| 127 | 32848 | 27933 | 27671 | 27760 | 26774 | 26544 | 80.80857282 |
| 128 | 42562 | 36442 | 36247 | 36214 | 35304 | 35217 | 82.74282224 |
| 129 | 47150 | 39867 | 39519 | 39462 | 38039 | 37274 | 79.05408271 |
| 130 | 43170 | 36543 | 36148 | 36274 | 34742 | 34368 | 79.61084086 |
| 131 | 48741 | 41764 | 41439 | 41505 | 40215 | 39978 | 82.02129624 |
| 132 | 55023 | 47097 | 46774 | 46755 | 45113 | 44690 | 81.22058048 |
| 133 | 49401 | 42263 | 41878 | 41869 | 40537 | 40349 | 81.67648428 |
| 134 | 55167 | 47723 | 47246 | 47284 | 44860 | 44539 | 80.73485961 |
| 135 | 61124 | 52402 | 52122 | 52114 | 50915 | 50654 | 82.87088541 |
| 136 | 51318 | 43955 | 43377 | 43476 | 40983 | 40878 | 79.65626096 |
| 137 | 40941 | 35004 | 34652 | 34573 | 33091 | 32716 | 79.91011456 |
| 138 | 23290 | 19969 | 19738 | 19699 | 18762 | 18564 | 79.7080292 |
| 139 | 41408 | 35594 | 35222 | 35211 | 33694 | 33314 | 80.45305255 |
| 140 | 38626 | 32528 | 32113 | 32195 | 30642 | 30585 | 79.18241599 |
| 141 | 50635 | 43308 | 42903 | 42965 | 40992 | 40756 | 80.4897798 |
| 142 | 47317 | 41095 | 40577 | 40731 | 38371 | 37708 | 79.69228818 |
| 143 | 60615 | 51837 | 51546 | 51671 | 50495 | 50154 | 82.74189557 |
| 144 | 57693 | 49439 | 49193 | 49198 | 48109 | 47523 | 82.37221153 |
| 145 | 49724 | 42972 | 42643 | 42457 | 40271 | 40120 | 80.68538332 |
| 146 | 51533 | 44430 | 44196 | 44241 | 43175 | 42733 | 82.92356354 |
| 147 | 37579 | 31978 | 31467 | 31492 | 29189 | 28781 | 76.58798797 |
| 148 | 51505 | 44093 | 43788 | 43741 | 42340 | 42111 | 81.76099408 |
| 149 | 65900 | 55880 | 55518 | 55563 | 53988 | 53720 | 81.51745068 |
| 150 | 7451 | 6395 | 6202 | 6266 | 5658 | 5640 | 75.69453765 |
| 151 | 42970 | 36533 | 36175 | 36258 | 34885 | 34495 | 80.2769374 |
| 152 | 51781 | 44274 | 43795 | 43837 | 41663 | 41396 | 79.94438114 |
| 153 | 56057 | 47721 | 47346 | 47462 | 45504 | 45161 | 80.5626416 |
| 154 | 54627 | 46456 | 45932 | 46034 | 43580 | 42652 | 78.07860582 |
| 155 | 38626 | 33032 | 32531 | 32727 | 30730 | 30405 | 78.71640864 |
| 156 | 49571 | 43035 | 42737 | 42780 | 41745 | 41589 | 83.8978435 |
| 157 | 50161 | 43331 | 42864 | 42842 | 40549 | 40317 | 80.37519188 |
| 158 | 42019 | 36243 | 35949 | 35950 | 34737 | 34564 | 82.25802613 |
| 159 | 45898 | 39083 | 38723 | 38769 | 36598 | 35278 | 76.86173689 |
| 160 | 32818 | 28007 | 27867 | 27854 | 27364 | 27157 | 82.75031995 |
| 161 | 57795 | 49271 | 49028 | 49027 | 47336 | 46840 | 81.0450731 |
| 162 | 32704 | 28012 | 27798 | 27722 | 26788 | 26683 | 81.58940802 |
| 163 | 53706 | 46186 | 45936 | 45996 | 45094 | 44848 | 83.50649834 |
| 164 | 47844 | 41194 | 40865 | 40868 | 39587 | 39230 | 81.99565254 |
| 165 | 56923 | 49099 | 48634 | 48797 | 46369 | 46056 | 80.90929853 |
| 166 | 36024 | 30720 | 30427 | 30470 | 29128 | 28812 | 79.98001332 |
| 167 | 47786 | 41491 | 41134 | 41184 | 39649 | 39080 | 81.78127485 |
| 168 | 43226 | 36914 | 36538 | 36635 | 35226 | 34744 | 80.37755055 |
| 169 | 50565 | 43887 | 43548 | 43556 | 42375 | 42000 | 83.06140611 |
